# Dangers of Spring

**Published:** 1859-05

**Authors:** 


### DANGERS OF SPRING.

About one fifth more persons die in New York city in May, than in November. After being pent up in the winter, it might be supposed that the ability to go out and exercise in the luscious air of spring time, would be productive of increased vigor and health of body, but this is simply not the case, as evidenced by the ably prepared and valuable reports of City Inspector Morton. This difference of mortality between the last month of spring, and the last month of fall, arises from causes which are under the control of the people or beyond; two of each will be mentioned. The natural causes are, 1st. The increased dampness of the asmosphere, proven by the fact that doors which shut easily in winter, do not do so in summer. 2nd. Nature takes away the appetite for meals, for heat giving food, in order to prepare the body for the increased temperature of summer. But two errors in practice at this time, interfere with wise nature's arrangements, and induce many and painful and dangerous diseases. First, the amount of clothing is diminished too soon. Second, the conveniences of fire in our dwellings are removed too early. All persons, especially children, old people, and those in delicate health, should not remove the thickest woolen flannel of mid-winter, nntil sometime in May, and then, it should be merely a change to a little thinner material.

Furnaces should not be removed, nor fire places and grates cleaned for tho summer, until the first of June; for a brisk fire in the grate is sometimes very comfortable in the last week in May; that may be a rare occurrence, but as it does sometimes take place, it is better to be prepared for it, than to sit shivering for half a day, with the risk to ourselves and children, of some violent attack of spring disease. By inattention to these things, four causes are in operation, to chill the body, and induce colds and fevers.

First, The dampness of the atmosphere in May.

Second, The striking falling off in appetite for meats and other "heating" food.

Third, The premature diminution of clothing.

Fourth, The too early removal of the conveniences of fire.

And when the very changing condition of the weather of May is taken into account, it is no wonder, that under the influence of so many causes of diminution of the temperature of the body, many fall victims to disease.

In November, the healthiest month in the year, we have put on our warmest clothing, we have kindled our daily fires, we have found a keen relish for substantial food, while the dampness of the atmosphere has been removed by the condensation of increasing cold. The wise will remember these things for a lifetime, and teach them to their children.

